# Hygromechanical Behavior of Polyamide 6.6: Experiments and Modeling

**DOI:** 10.3390/polym15163387

**Published:** 2023-08-12

**Authors:** Paul Wetzel, Anna Katharina Sambale, Kai Uhlig, Markus Stommel, Benjamin Schneider, Jan-Martin Kaiser

**Affiliations:** 1 Component Design, Reliability and Validation Polymers, Corporate Sector Research and Advance Engineering, Robert Bosch GmbH, Robert-Bosch-Campus 1, 71272 Renningen, Germany; 2 Institute of Polymer Materials, Leibniz-Institut für Polymerforschung Dresden e.V., Hohe Str. 6, 01069 Dresden, Germany; sambale@ipfdd.de (A.K.S.); uhlig@ipfdd.de (K.U.); stommel@ipfdd.de (M.S.); 3 Chair of Polymer Materials, Institute of Materials Science, Technical University Dresden, 01062 Dresden, Germany

**Keywords:** PA66, water sorption, diffusion, moisture-induced swelling, CME, plasticization

## Abstract

This paper investigates water absorption in polyamide 6.6 and the resulting hygroscopic swelling and changes in mechanical properties. First, sorption and swelling experiments on specimens from injection molded plates are presented. The observed swelling behavior is dependent on the melt flow direction of the injection molding process. Additionally, thermal analysis and mechanical tensile tests were performed for different conditioning states. The water sorption is accompanied by a decrease in the glass transition temperature and a significant reduction in stiffness and strength. Next, a sequentially coupled modeling approach is presented. A nonlinear diffusion model is followed by mechanical simulations accounting for swelling and concentration-dependent properties. For the mechanical properties, the notion of a “gap” temperature caused by the shift of the glass transition range due to water-induced plasticization is employed. This model enables the computation of local moisture concentration fields and the resultant swelling and changes in stress–strain behavior.

## 1. Introduction

Polyamides are widely used for industrial applications due to their good processing characteristics and mechanical properties [1,2]. Their ability to absorb water is a well-known effect [3,4] that strongly impacts the material behavior and performance. Water molecules bound between the polymer chain segments lead to an increase in molecular mobility and spacing between the polymer chains [1,5]. This results in moisture-induced swelling and plasticization of the material. The water-induced plasticizing effect manifests in a shift of the glass transition range toward lower temperatures and significant changes in mechanical properties [2,6,7]. These properties can vary locally in a plastic part as inhomogeneous moisture concentration fields emerge until the complete part is in equilibrium with its environment [8].

The description of moisture sorption and its effects on polyamides is discussed in the literature. A detailed review on the effect of water in polyamides is given in [9]. In [5,10], a diffusion model has been developed and validated by reconstructing predicted moisture gradients with CT scan measurements. In [8], the model is coupled to elastic properties and applied to mechanical tests with inhomogeneous moisture distributions. Further studies, such as [1,11], focus on different modeling approaches for the water diffusion in polyamides. In [12,13,14,15], the sorption and swelling behavior of polymer films in contact with water and water vapor is discussed. The influence of moisture on viscoelastic properties is highlighted in [16,17,18,19]. First approaches to model the shift of the glass transition range due to moisture uptake are presented in [20,21]. In [22], the shift of the glass transition range in the presence of inhomogeneous moisture distributions is studied. Mechanical models for polyamides in contact with water often rely on the notion of a “gap” temperature linked to the shift of the glass transition temperature due to plasticization. This approach has been applied to plastic yield stress changes [2,7] and deformation behavior [23,24] as well as viscoelastic properties [25,26,27] for various polyamide grades.

The aim of the present paper is to investigate moisture diffusion in polyamide 6.6, hygroscopic and thermal expansion, and changes in elastic–plastic properties due to the presence of water. To this end, the experiments presented in Section 2 were performed, and the chosen modeling approach is discussed in Section 3.

## 2. Experiments

### 2.1. Sample Preparation

For the present study plates with an area of 80 × 80 mm^2^ and three different thicknesses, namely 1, 2 and 4 mm, have been injection molded on an Engel e-motion 220 T (Engel Austria GmbH, Schwertberg, Austria) using the commercial polyamide 6.6 grade BASF Ultramid A3K (uncolored). Melt processing and mold temperature were set to 290 °C and 90 °C, respectively. The initial water content of the granulate and the molded plates was examined using KARL FISCHER titration (DIN EN ISO 15512), each revealing a water mass fraction of less than 0.1%. To prevent moisture sorption, the plates were stored in vacuum-sealed aluminum bags until the test specimens were removed by milling and tested.

### 2.2. Sorption Experiments

#### 2.2.1. Setup

In order to characterize the sorption behavior, plate-shaped specimens of (20×15×1,2,4) ${\mathrm{mm}}^{3}$ were used. From each molded plate, five specimens were cut from the center region. Before conditioning and testing, the milled samples were additionally dried in a vacuum furnace at 50 °C (DIN EN ISO 62) [28]. Subsequently, separate batches with dry specimens of different thickness were conditioned according to the parameters given in Table 1 using a water bath (Memmert WNB 14, Schwabach, Germany) and a climate chamber (ESPEC LHL 114, Osaka, Japan). During the sorption process, the specimens were weighed recurrently using a laboratory balance (Mettler AE200, ±0.1mg, Greifensee, Switzerland) until the relative mass gain remained approximately constant.


(1) $$M=\frac{m-m_{\mathrm{dry}}}{m_{\mathrm{dry}}}$$

#### 2.2.2. Results

**Figure 1:**
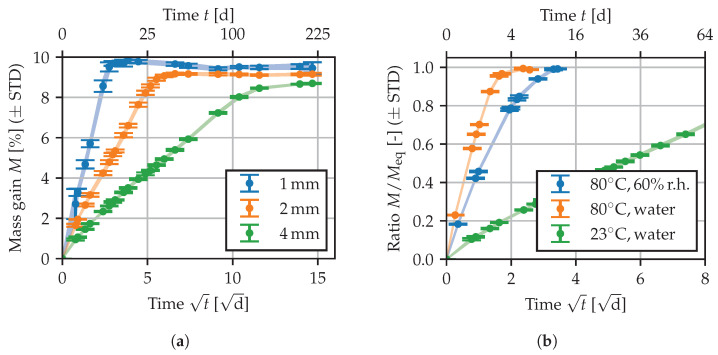
Sorption curves: (**a**) for different specimens in 23 ${}^{{}^{\circ}}C$ water, (**b**) normalized for 4 mm specimens at different ambient conditions. Shaded areas between the error bars serve as a guide to the eye.

In Figure 1, a selection of the recorded sorption curves is given to illustrate observed effects. The complete data are depicted in the modeling Section 3.2 along with the prediction of the chosen diffusion model. A square root time scale is used, such that the initial part of the sorption curves is approximately linear. In Figure 1a, the sorption curves for immersion in demineralized water at 23 °C are depicted. In general, the thicker specimens tend to demonstrate slightly lower mass gains when reaching equilibrium, and the sorption behavior is significantly slower, leading to longer storage times. In the following, the equilibrium mass gain $M_{\mathrm{eq}}$ is defined as the mean of the last two points of all curves, i.e., averaging over all specimens of different thickness. Discrepancies between specimens of different thickness may be attributed to morphological differences caused by the injection molding process. For all conditioning states, the measure $M_{\mathrm{eq}}$ is listed in Table 1. As expected, less water is absorbed at lower ambient humidity, and for immersion in water, increased temperatures lead to a slight decrease in $M_{\mathrm{eq}}$. Figure 1b depicts normalized sorption curves of 4 mm specimens for different conditioning states. At lower temperature as well as for lower levels of ambient humidity, longer storage times are needed to reach equilibrium.

The storage at 70 °C/62%r.h. (DIN EN ISO 1110, $M_{\mathrm{eq}}=3.2\%$) or at 80 °C/60%r.h. ($M_{\mathrm{eq}}=3.0\%$) represents an accelerated conditioning that is used to attain a similar equilibrium moisture content as in standard atmosphere (23 °C/50%r.h.) [29]. Additionally, a separate set of 2 mm specimens has been stored in standard atmosphere, yielding $M_{\mathrm{eq}}=$ 3.0% after 8 months of storage. As in [30,31], the conditioning according to ISO 1110 has been found to lead to a slight “over-conditioning”, such that additional time is needed to adapt to 23 °C/50%r.h. In the following, “23/50” indicates the use of such an accelerated conditioning method accompanied by further storage at 23 °C/50%r.h.

### 2.3. Swelling Measurements

#### 2.3.1. Setup

To characterize the swelling behavior, the specimen dimensions were measured immediately before as well as after the sorption experiment. This was performed only for 80 °C/60%r.h., 80 °C/90%r.h., and 60 °C water using the 1, 2 and 4 mm specimens. The dimension measurement itself took place at 23 °C after the specimens had been wiped dry with a lint-free cloth and briefly adapted to room temperature. In order to evaluate whether the hygroscopic expansion takes place isotropically or anisotropically as suggested in [5], two independent measuring methods have been used. At first, the length, width and thickness of the samples was recorded using a digital outside micrometer (Mitutoyo Digimatic, ±1μm). Secondly, the density and the volume of the samples were measured with the buoyancy method (Mettler ME 33360) by weighing the samples in air and during brief immersion in demineralized water. In the following, *x* denotes the sample length corresponding to the mold flow direction, *y* denotes the sample width perpendicular to the flow within the plane and *z* denotes the thickness direction. From the dimensional changes in *x*, *y* and *z*, the hygroscopic swelling strains $\varepsilon =L_{1}\;/\;L_{0}-1$ are calculated. The indices 0 and 1 refer to the dry state at room temperature and the state after completion of the sorption experiment and briefly cooling down to room temperature, such that a homogeneous moisture distribution within the specimen is assumed, respectively. For validation purposes, the equivalent isotropic strain

(2) $$\varepsilon_{\mathrm{iso}}={\left(\left(1+\varepsilon_{\mathrm{xx}}\right)\;\left(1+\varepsilon_{\mathrm{yy}}\right)\;\left(1+\varepsilon_{\mathrm{zz}}\right)\right)}^{\frac{1}{3}}-1$$

is compared to the values

(3) $${\tilde{\varepsilon }}_{\mathrm{iso}}={\left(\frac{V_{1}}{V_{0}}\right)}^{\frac{1}{3}}-1$$

obtained from measuring the volume change using the buoyancy method.

#### 2.3.2. Results

**Figure 2:**
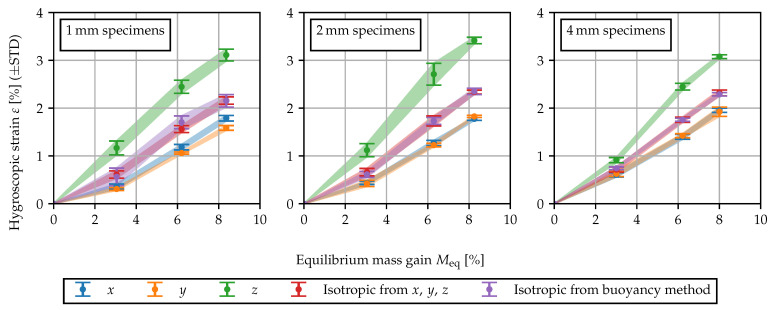
Hygroscopic swelling measured tactilely in three directions as well as volumetrically by means of the buoyancy method. The swelling strain is plotted against the mass gain for equilibrium at 80 °C/60%r.h. ($M_{\mathrm{eq}}=3.0\%$), 80 °C/90%r.h. ($M_{\mathrm{eq}}=6.2\%$) and in 60 °C water ($M_{\mathrm{eq}}=8.2\%$). Shaded areas between the error bars serve as a guide to the eye.

In Figure 2, the swelling results are shown. Firstly, the results indicate that the expansion behavior is fairly linear with respect to the equilibrium moisture content. Secondly, while the dimensional changes in *x*- and *y*-direction are similar, the expansion in thickness direction is significantly more pronounced. The observed anisotropy of the swelling behavior is in agreement with the findings presented in [5]. The volumetric measurements and the equivalent isotropic strain from the measurements in *x*, *y* and *z* are in good agreement. Within the thicker 4 mm specimens, the anisotropic swelling behavior is less pronounced. The observed anisotropy may be attributed to the complex morphology caused by the injection molding process. Two possible explanations are as follows: As the water molecules are bound between the polymer chains, a high degree of molecular orientation may cause anisotropic moisture expansion locally within the specimen. Furthermore, the skin-core effect may lead to a layered structure and hence deviating sorption and swelling properties across the specimen thickness. Depending on the direction in which expansion is measured, these deviating properties act in series or in parallel, causing a global anisotropy of the specimen. The heterogeneous and anisotropic morphology through the cross-section of injection molded polyamide specimens is reported upon in [32,33].

**Figure 3:**
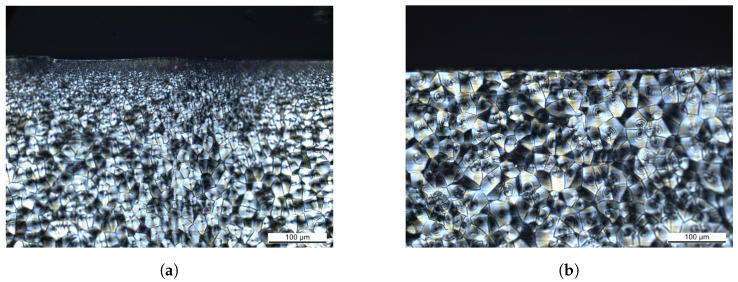
Circular polarized light microscopy on specimens from injection molded 4 mm plates: (**a**) original 4 mm specimen with semi-crystalline structure and increasing spherulite sizes toward the core, (**b**) 2 mm core specimen with large spherulites dominating.

To further investigate this effect, core specimens of 2 mm thickness have been machined from the 4 mm specimens by means of grinding and polishing. As 1 mm each from the bottom and the top side has been removed, the specimens represent the core layer of the injection molded plate. In Figure 3, circular polarized light microscopy images of microtome sections in the *y*-*z* plane are shown. For the 4 mm specimen, the observed morphology consisting of a transparent skin layer and increasing spherulite sizes toward the core (Figure 3a) is typical for injection molded polyamide parts [33]. The morphology of the core specimen (Figure 3b) is characterized by larger spherulites from the core layer.

**Figure 4:**
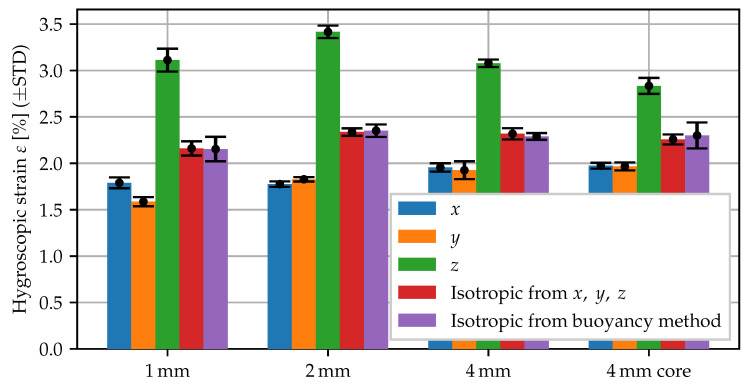
Swelling behavior of specimen types with different morphology: 1, 2 and 4 mm plate and 2 mm core region of 4 mm plate.

Using the core specimens, the swelling measurement for conditioning in 60 °C water has been repeated. The swelling strains are summarized in Figure 4. By comparing the 1,2 and 4 mm specimens and the core specimens, it becomes apparent that the anisotropic behavior is quite robust. The degree of anisotropy, i.e., how much the expansion in thickness direction differs, is slightly less pronounced in the core specimens, yet still clearly observable, whereas the observed volume change remains fairly constant.

### 2.4. Thermal Analysis

#### 2.4.1. Setup

To characterize the thermal expansion behavior and the glass transition range, thermomechanical analysis (TMA) and differential scanning calorimetry (DSC) measurements have been conducted.

The TMA measurements (Q400, TA Instruments, New Castle, DE, USA) were carried out on (4×4×4) ${\mathrm{mm}}^{3}$ cube-shaped specimens cut from the injection molded plates. The conducted experiments are summarized in Table 2. For a dry specimen, separate measurements in the *x*-, *y*- and *z*-direction have been conducted to examine the direction dependence, and further conditioning states have been tested to examine the effect of moisture. For each test, the results from a second heating run at 5 K min after an initial heating run at 10 K min were used.

The DSC measurements (Q2000, TA Instruments) were carried out on samples (approx. 6 mg) cut from dry and conditioned 1 mm plates using a scalpel. The water-induced shift in $T_{g}$ was evaluated using the half-step height method. For this, the results from the first heating run at 10 K min were used. An overview of the conducted measurements is given in Table 3. The start and end temperatures have been chosen with respect to the expected range of $T_{g}$. The DSC has been calibrated using sapphire and indium standards, and a liquid nitrogen cooling system (LNCS) and standard aluminum sample pans with lids were used.

#### 2.4.2. Results

**Figure 5:**
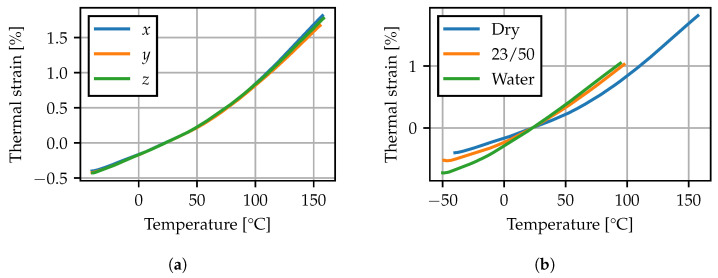
TMA: (**a**) dry specimen in *x*-, *y*- and *z*-direction, (**b**) specimens with different conditioning states in *z*-direction.

In Figure 5a, the TMA results for the dry specimen are given. The thermal strain is calculated with respect to the distance recorded at 23 °C. Contrary to the hygroscopic swelling, the thermal expansion behavior is similar for all three directions. Additionally, an increase in the thermal expansion slope can be observed at elevated temperatures, which is associated with passing the glass transition range. Moreover, the effect of increased moisture contents has been analyzed. In Figure 5b, the thermal expansion behavior in *z*-direction of further specimens conditioned to 23/50 and in 60 °C water is compared to the results from the dry sample. The altered expansion behavior is superimposed by effects due to redrying of the sample during heating. Comparing the conditioned specimen weight before and after the TMA experiment, a significant weight loss of 0.6% for the 23/50 specimen and 2% for the water bath specimen was observed. Consequently the measurement does not correspond to a known homogeneous moisture content within the test specimen. Nevertheless, the results indicate that the conditioning state significantly impacts the thermal expansion behavior. At elevated moisture contents, the thermal expansion slope increases at lower temperatures, suggesting a shift of the glass transition range.

**Figure 6:**
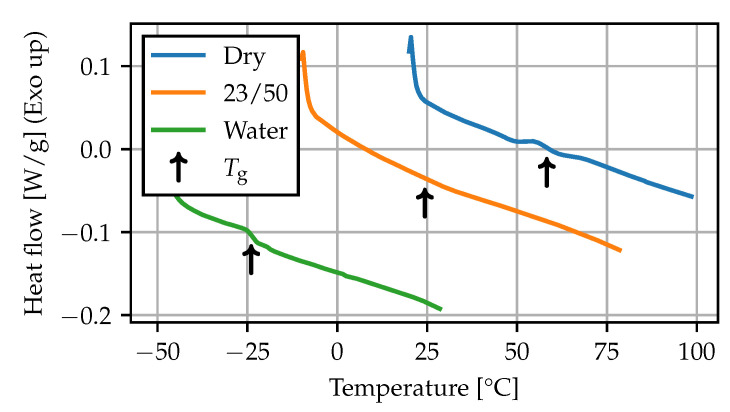
DSC: thermograms (10 K min^−1^).

For the DSC measurements, the thermograms during the first heating run at 10 K $\min^{-1}$ are shown in Figure 6, and the recorded $T_{g}$ values are summarized in Table 4. The measurement for the dry state falls within the lower end of the range given in the literature on PA66 [4,11,34]. The observed shift of more than 80 K is in agreement with results found in the literature on plasticization of PA6 [7,22,35].

### 2.5. Tensile Tests

#### 2.5.1. Setup

In order to characterize the effect of moisture sorption on the mechanical behavior, tensile tests have been conducted at different temperatures $\left(0,\;23,\;60,\;80\;{}^{{}^{\circ}}C\right)$ and conditioning states (dry, 23/50, 60 °C water) on a Zwick 1456 universal testing machine. The tensile specimens (DIN EN ISO 8256, type 3) were milled from the center of the 2 mm plates and are oriented in the flow direction of the molding process. At all temperatures, the strain measurement was conducted using a contact extensometer (Zwick multiXtens, Shanghai, China), and the test speed was 5 mm min^−1^. To analyze the lateral contraction, the measurements at room temperature have been repeated using digital image correlation (GOM ARAMIS 3D). The dry specimens were stored in a desiccator until testing to avoid moisture sorption, as the laboratory was regulated to 23 °C, 50%r.h. The 23/50 specimens were stored in the laboratory and tested briefly after being put into the temperature chamber to avoid redrying. After conditioning, the water bath specimens were temporarily stored in water at the corresponding testing temperature until immediately before clamping and testing. For each condition, three specimens were tested.

#### 2.5.2. Results

**Figure 7:**
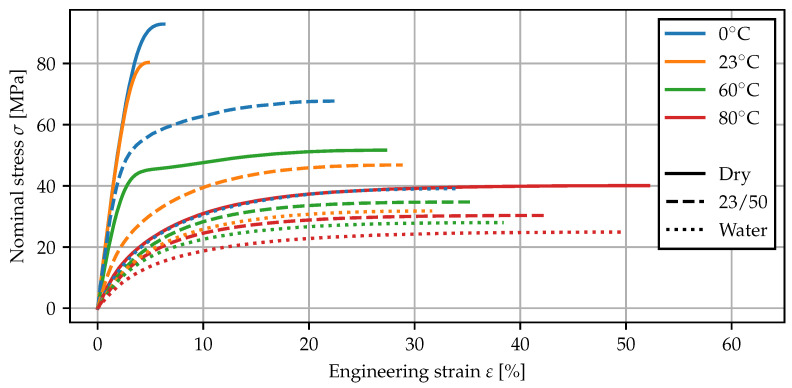
Tensile tests at different temperatures and conditioning states. The mean curves from three separate measurements are computed and cut off at the maximum nominal stress.

In Figure 7, the mean stress–strain curves for the tests using the contact extensometer are plotted up to the maximum nominal stress. As both an increase in temperature as well as the presence of water increase the molecular mobility [2], the corresponding stress–strain curves exhibit a reduced Young’s modulus and an overall more ductile behavior. In particular, the measurements of dry specimens at 80 °C and water bath specimens at 0 °C show almost the same behavior, suggesting similarities between the effects of temperature and moisture sorption. For the tests at room temperature, the measured Poisson’s ratios (GOM) and Young’s moduli (multiXtens) are given in Table 5. The elasticity parameters have been fitted using linear regression in the interval from 0 to 0.5% strain. After conditioning in water, the stiffness is reduced by a factor of more than four, while the Poisson’s ratio tends toward 0.5.

## 3. Modeling

### 3.1. Modeling Approach

The model developed here aims to describe the moisture transport in the polymeric material as well as swelling and effects on mechanical properties. The local moisture concentration *c* is defined as the species density [36], which describes the mass of the absorbed water per unit reference volume. For any subregion $\Omega_{R}$ of the undeformed body $B_{R}$, the mass of water molecules absorbed by the polymer is given by

(4) $$m_{\mathrm{water}}=\int_{\Omega_{R}}cdV\;.$$

The density of the polymer in the dry state $\rho_{R}^{\mathrm{dry}}$ is assumed to be constant for the material at hand, such that $c_{\mathrm{eq}}=\rho_{R}^{\mathrm{dry}}M_{\mathrm{eq}}$, when equilibrium is reached. Its value $\rho_{R}^{\mathrm{dry}}=1.13\;g\;{\mathrm{cm}}^{3}$ is taken from the supplier data sheet [37] and has been confirmed by the buoyancy measurements. The evolution of the concentration field is modeled as a diffusion problem, neglecting possible dependencies on the state of stress or deformation. Subsequently, the computed concentration field can be mapped to mechanical simulations, which account for hygroscopic swelling and moisture-dependent mechanical properties. The sequentially coupled model is implemented in Abaqus FEA. For the mechanical response of the material, an elastic–plastic modeling approach is used. The geometrically linear model is based on the additive decomposition of the strain tensor

(5) $$\varepsilon =\underset{\varepsilon^{m}}{\underset{︸}{\varepsilon^{\mathrm{el}}+\varepsilon^{\mathrm{pl}}}}+\underset{\varepsilon^{*}}{\underset{︸}{\varepsilon^{\mathrm{hy}}+\varepsilon^{\mathrm{th}}}}$$

into a mechanical strain $\varepsilon^{m}$, containing elastic and plastic contributions, and the expansion strain $\varepsilon^{*}$ that is caused by hygroscopic and thermal expansion and does not contribute to the evolution of internal stresses.

### 3.2. Moisture Diffusion

**Figure 8:**
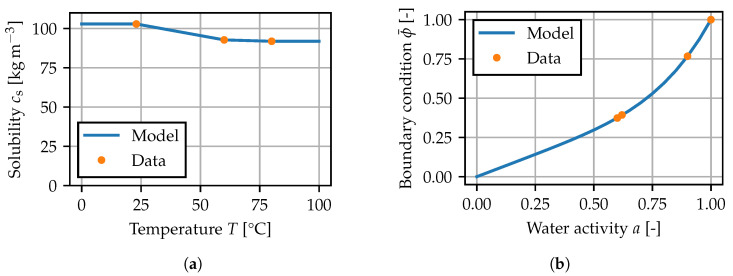
Equilibrium relation for the diffusion problem: (**a**) temperature-dependent solubility parameter, (**b**) boundary condition for the normalized concentration.

The diffusion problem is governed by the balance of mass for the diffusing phase

(6) $$\dot{c}=-\vec{\nabla }\;\cdot \;\vec{J}$$

along with a constitutive equation for the mass flux $\vec{J}$ and appropriate initial and boundary conditions. In addition, the normalized concentration

(7) $$\phi =\frac{c}{c_{s}}$$

is introduced and chosen as the degree of freedom in the finite element model [38]. The solubility $c_{s}\left(T\right)$ is defined as the equilibrium concentration after immersion in water. This is interpreted to be the maximum amount of water the material can absorb at a given temperature. The measured data, between which $c_{s}\left(T\right)$ is interpolated linearly, are plotted in Figure 8a.

For the boundary condition $\phi =\bar{\phi }$, it is assumed that $\bar{\phi }\left(a\right)$ can be given as a function of the water activity *a* [1,11], such that the temperature dependence in

(8) $$c_{\mathrm{eq}}\left(T,\;a\right)=c_{s}\left(T\right)\;\bar{\phi }\left(a\right)$$

is only governed by the solubility. For the model, the relationship

(9) $$\bar{\phi }\left(a\right)=k_{1}a+\left(1-k_{1}\right)a^{k_{2}}$$

is chosen. In contact with air, *a* is taken as equal to the relative humidity RH [39] at the given ambient temperature, and immersion in demineralized water corresponds to a=1. This expression is similar to the approaches in [1,11,40,41] and involves a linear part, that is related to Henry’s law, and a nonlinear contribution. The model fitted to the given data is shown in Figure 8b.

The constitutive equation for the mass flux is given by

(10) $$\vec{J}=-c_{s}\left(T\right)\;D\left(T,\;c\right)\;\vec{\nabla }\phi \;.$$

In the following, a linear and a nonlinear model are considered.

**Figure 9:**
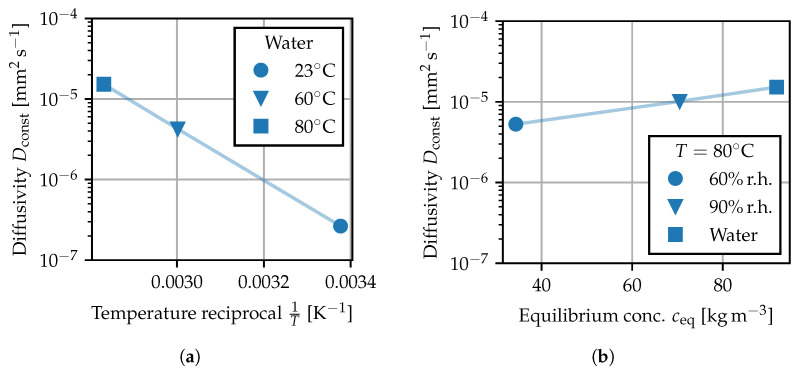
Constant diffusivity fitted to sorption experiments: (**a**) by temperature, (**b**) by ambient humidity. Shaded lines are used to connect the values $D_{\mathrm{const}}$ as a guide to the eye.

For the linear model, the diffusivity does not depend on the concentration *c*. Assuming constant temperature and a constant diffusivity $D=D_{\mathrm{const}}$ within the specimen, the linear heat equation

(11) $$\dot{c}\left(\vec{x},\;t\right)=D_{\mathrm{const}}{\vec{\nabla }}^{2}c\left(\vec{x},\;t\right)$$

is recovered. For a cuboid specimen with the boundary conditions ${c|}_{x_{i}=\pm MML_{i}\;/\;2}=c_{\mathrm{eq}}$ and the initial condition ${c|}_{t=0}=c_{0}=0$, the integral mass gain is given by the infinite series

(12a) $$\frac{c_{\mathrm{eq}}-\rho_{R}^{\mathrm{dry}}M\left(t\right)}{c_{\mathrm{eq}}-c_{0}}=\prod_{i=1}^{3}f\left(\eta =\frac{4Dt}{L_{i}^{2}}\right)$$

with

(12b) $$f\left(\eta \right)=\frac{8}{\pi^{2}}\sum_{n=0}^{\infty }\frac{1}{{\left(2n+1\right)}^{2}}\exp\left(-\frac{\pi^{2}{\left(1+2n\right)}^{2}\eta }{4}\right)$$

and can be compared to the recorded measuring points [42,43,44]. Using least squares, a reference value for $D_{\mathrm{const}}$ is fitted to each sorption experiment from Table 1. In Figure 9, the fitted values for $D_{\mathrm{const}}$ are given for the water bath experiments at different temperatures as well as for the experiments at 80 ${}^{\circ }C$ and deviating ambient humidity. For each sorption experiment, $D_{\mathrm{const}}$ describes how quickly equilibrium is reached depending on temperature and ambient humidity. The values in Figure 9a show that the diffusivity strongly depends on temperature. The observed temperature dependence is in good agreement with the Arrhenius law and is attributed to an increased mobility of the polymer chains. The reference values for $D_{\mathrm{const}}$ in water are in the same order of magnitude as the data reported in [4] for a PA66 grade at the same test temperatures. The values in Figure 9b reveal that the rate at which equilibrium is reached also demonstrates a less pronounced dependence on the level of ambient humidity, i.e., the equilibrium concentration toward which the specimen tends. This effect is modeled by making the diffusion coefficient depend on the local concentration field D=D(c). This approach makes the diffusion problem non-linear or non-Fickian [1], such that Equations (11) and (12) no longer hold and the sorption behavior must be computed numerically. The increase in diffusivity at elevated local moisture contents can be explained by the water-induced plasticizing effect. Similar to the effects of elevated temperature, water molecules bound between the polymer chain segments lead to an increase in molecular mobility and spacing between the polymer chains [1,5], suggesting that the water molecules can move more freely within in the base material.

**Figure 10:**
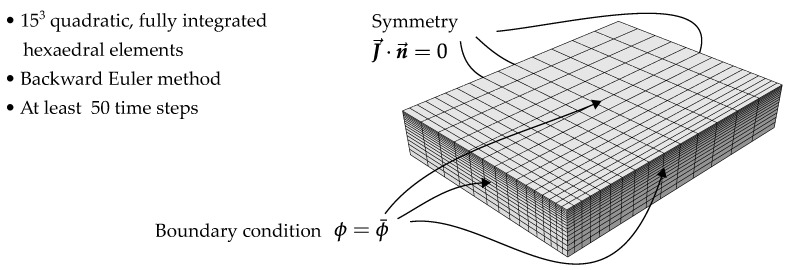
FE model for plate-shaped specimen with symmetry in all three directions. For the boundary condition, $\vec{n}$ denotes the normal vector.

**Figure 11:**
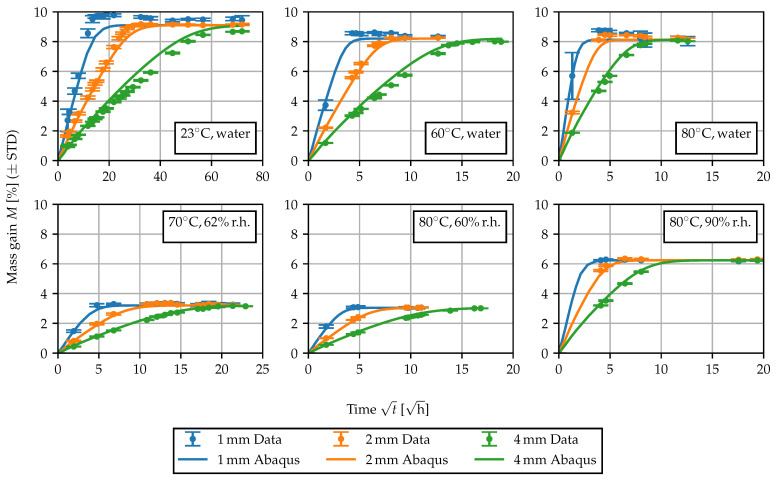
Comparison of the experimentally and numerically obtained sorption curves.

For the nonlinear model, the empirical expression

(13) $$D\left(T,\;c\right)=D_{0}\;\exp\left(k\left(\frac{c}{c_{s}\left(T\right)}-1\right)\right)\;\exp\left(-\frac{E_{a}}{RT}\right)$$

is chosen to model the concentration-dependent diffusivity. Motivated by the behavior of $D_{\mathrm{const}}$, the temperature dependence is modeled using an Arrhenius law [1,5] and the increase in diffusivity at elevated moisture concentrations is described with an exponential law. The unknown parameters are $D_{0}$, *k* and the activation energy $E_{a}$. *R* is the universal gas constant. As for the previous identification of $D_{\mathrm{const}}$, the parameters of the nonlinear model were fitted to the experimentally determined sorption curves using least squares. While for the linear diffusion problem the infinite series Equation (12) was used, for the model with concentration-dependent diffusivity, the model prediction was computed directly in Abaqus FEA using a three-dimensional model of the plate-shaped specimens. The mesh for the 4 mm specimens and some further details are shown in Figure 10. For the identification of the parameters $D_{0}$, *k* and $E_{a}$, all sorption curves were taken into account simultaneously, i.e., the sum of the mean squared error of all curves was minimized. The comparison between the predicted mass gain curves and the experimental data is shown in Figure 11. Overall, the experimentally determined sorption behavior is captured well, as the rate of the sorption process can be predicted, depending on temperature and ambient humidity. Nevertheless, the behavior of the specimens of different thickness can only be described as an average. Particularly for the sorption experiment in 23 ${}^{\circ }C$ water, the thicker specimens demonstrate a slightly reduced equilibrium concentration, and the rate at which equilibrium is achieved is disproportionately slower. These differences may be attributed to differences in the morphological structure [5,33] due to the injection molding processes. Furthermore, water-induced changes in the morphological structure can occur, since the water is able to induce recrystallization and solid state phase transformations in the crystalline phase of the material [45].

### 3.3. Hygroscopic Expansion

In order to model the expansion behavior, it is necessary to distinguish between hygroscopic and thermal expansion. In the following, the expansion strain $\varepsilon^{*}$ is assumed to be a function of temperature and concentration, i.e., the expansion due to temperature and humidity is interpreted as a fully reversible phenomenon. For the reference state, $T_{\mathrm{ref}}=23$ °C and $c_{\mathrm{ref}}=0$, $\varepsilon^{*}$ is assumed to vanish. The hygroscopic swelling has been measured at room temperature and can be defined as

(14) $$\varepsilon^{\mathrm{hy}}\left(c\right)=\varepsilon^{*}\left(T_{\mathrm{ref}},\;c\right)\;.$$

The measured deformations shown in Figure 2 after conditioning to states of equilibrium are assumed to be solely due to swelling and are interpreted as $\varepsilon^{\mathrm{hy}}\left(c\right)$. As the observed behavior is approximately linear with respect to the moisture content, a constant coefficient of moisture expansion (CME)

(15) $$\varepsilon^{\mathrm{hy}}=\beta \;c$$

is fitted by means of linear regression. To capture the direction dependence, orthotropic values can be provided for $\beta_{\mathrm{xx}}$, $\beta_{\mathrm{yy}}$ and $\beta_{\mathrm{zz}}$. Alternatively, an isotropic model can be used, such that the volume change is captured. The CME values for the 2 mm specimens are given in Table 6.

### 3.4. Thermal Expansion

The remainder

(16) $$\varepsilon^{\mathrm{th}}\left(T,\;c\right)=\varepsilon^{*}\left(T,\;c\right)-\varepsilon^{*}\left(T_{\mathrm{ref}},\;c\right)$$

is interpreted as thermal expansion and modeled using a “mean” or secant form of the thermal expansion coefficient (CTE)

(17) $$\varepsilon^{\mathrm{th}}=\alpha \left(T,\;c\right)\;\left(T-T_{\mathrm{ref}}\right)\;.$$

Herein, the tensor α=αI is assumed to be isotropic (compare Figure 5a).

The experimental results presented in Section 2.4 indicate that the moisture concentration has an influence on the thermal expansion behavior. Nevertheless, reliable measurements for elevated moisture concentrations can only be obtained by controlling the sample’s humidity environment. Here, as a further simplification, the concentration dependence of the thermal expansion behavior is dropped, and the mean of the direction-dependent measurements for the dry state is used. Values for the secant CTE sampled at exemplary temperature points are given in Table 7.

### 3.5. Mechanical Deformation

The moisture- and temperature-dependent deformation behavior is described using an elastic–plastic modeling approach. Further causes for nonlinearities in the uniaxial stress–strain behavior, such as nonlinear elastic deformations, viscous effects or damage, are not dealt with in the present work. The elastic part is governed by the Young’s modulus *E* and the Poisson’s ratio ν, and a simple von Mises plasticity model with isotropic hardening [46] is used. Two parameters characterizing the plastic response are the initial yield stress $\sigma_{y0}$, i.e., the onset of nonlinear stress–strain behavior and the nominal stress maximum $\sigma_{\infty }$. As in [2], the initial yield stress is defined as the point where stress–strain plot deviates by 5% from linear behavior.

To model the influence of the local moisture content, the notion of a “gap” temperature is employed. This concept is based on the idea that both the presence of water as a plasticizer as well as elevated temperatures lead to an increase in mobility of the polymer chains, such that their effect on mechanical properties can be treated in a similar manner. The temperature shift ΔT(c) is interpreted as the reduction in the glass transition temperature $T_{g}$ and can either be obtained from the shift of the mechanical properties or from separate $T_{g}$ measurements. This modeling approach has previously been applied in the literature on plastic yield stress changes [2,7] and deformation behavior [23,24], as well as viscoelastic properties [25,26,27] for various polyamide grades.


(18) $$\tilde{T}=T-\Delta T\left(c\right)=T-\left(T_{g}\left(c\right)-T_{g}^{\mathrm{dry}}\right)$$

**Figure 12:**
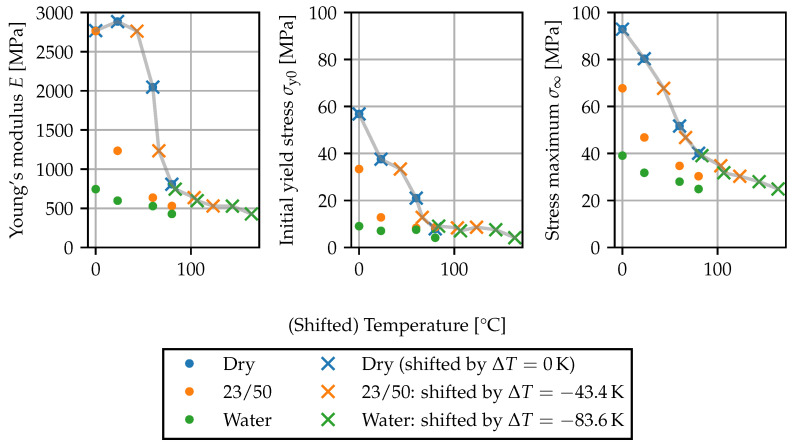
Temperature-moisture equivalence of parameters: Parameters plotted against temperature *T* for different moisture contents and parameters plotted against shifted temperature $\tilde{T}=T-\Delta T\left(c\right)$ for elevated moisture contents. The gray lines connect the shifted points as a guide to the eye.

In Figure 12, the gap temperature concept is applied to the parameters *E*, $\sigma_{y0}$ and $\sigma_{\infty }$ characterizing the tensile test curves from Section 2.5. The shift ΔT(c) was fitted to the data for the Young’s modulus *E*, such that the curves corresponding to the dry state, 23/50 and conditioning in water are superposed. The same shift values are applied to the initial yield stress $\sigma_{y0}$ and the nominal stress maximum $\sigma_{\infty }$. Overall, the shift leads to a smooth behavior of the mechanical properties, implying that $\tilde{T}$ is a suitable variable for interpolating the stress–strain curves depending on temperature and local moisture content.

**Figure 13:**
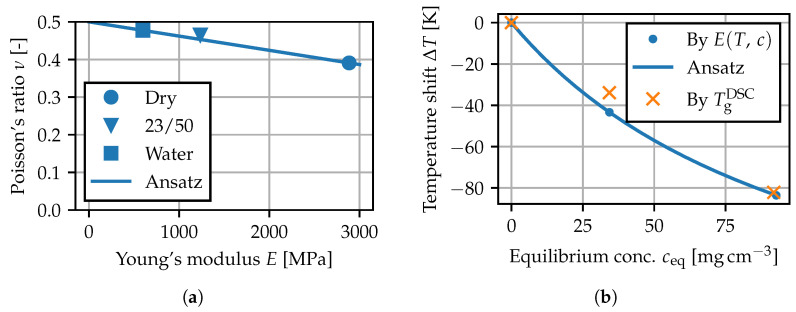
Parameters for the mechanical model: (**a**) prediction of the Poisson’s ratio, (**b**) temperature shift for concentration-dependent mechanical properties.

The Poisson’s ratio ν is assumed to depend linearly on *E*, such that the bulk modulus corresponding to *E* and ν measured in the dry state at 23 °C remains constant for all *T* and *c*. In Figure 13a, this model, for which ν tends toward 0.5 as *E* is decreased by temperature and moisture, is compared to the measurements at room temperature. The approach is similar to approximation formulas such as according to Meder and Giencke [3,5,47].

To model the temperature shift ΔT as a function of *c*, an expression based on the Kelley–Bueche equation [6,20] for the change in $T_{g}$ is used:

(19) $$\Delta T=\frac{\chi T_{g\Delta }}{\chi +A\;(1-\chi )}\;\mathrm{with}\;\chi =\frac{c}{c+\rho_{R}^{\mathrm{dry}}}\;.$$

The variables *A* and $T_{g\Delta }$ are the unknown parameters, and χ denotes the mass fraction of water. The parameters are fitted to the ΔT values found for the conditioning states in Figure 12. In Figure 13b, the prediction of the temperature shift and the $T_{g}$ drop measured by means of DSC (Table 4) are compared. The differences are 1.0 K for conditioning in water and 9.5 K at 23/50, overall indicating that the shift in mechanical properties and the $T_{g}$ shift are closely connected.

## 4. Conclusions

In this study, the water sorption in polyamide 6.6 as well as the resultant swelling and changes in mechanical properties were characterized by experiments at various temperatures and modeled using finite element software. The main results are listed as follows:

- In order to model the water sorption behavior based on experiments on specimens from injection molded plates, a nonlinear diffusion model was employed. The chosen model is in good agreement with the experiments at various temperatures and levels of ambient humidity.
- The moisture-induced swelling was measured in different directions, as well as volumetrically for multiple equilibrium moisture contents and has been found to be anisotropic. Additionally, specimens from the core layer of the injection molded plates were machined and tested to investigate the influence of the specimen morphology on the anisotropy of the swelling process. In contrast, the thermal expansion behavior has been identified as isotropic.
- The changes in mechanical properties, derived from tensile tests at various temperatures and conditioning states, are described by an apparent temperature shift that depends on the local moisture content. The shift is linked to the drop in $T_{g}$ due to the water-induced plasticizing effect.

The effect of local moisture distributions can be modeled in a sequentially coupled manner. Following a mass diffusion analysis, the computed concentration field can be used to account for hygroscopic swelling and changes in mechanical properties.

## Figures and Tables

**Table 1:** Conditioning parameters used for characterizing the sorption behavior.

Ambient Humidity	Temperature (°C)	Mean Equilibrium Mass Gain $M_{\mathrm{eq}}$ (%)
Water	23	9.1
Water	60	8.2
Water	80	8.1
90%r.h.	80	6.2
62%r.h.	70	3.2
60%r.h.	80	3.0

**Table 2:** TMA experiments: overview.

Conditioning	$M_{\mathrm{eq}}$ (%)	Direction	$T_{\min}$ (°C)	$T_{\max}$ (°C)
Dry	0.0	*x*	−40	160
Dry	0.0	*y*	−40	160
Dry	0.0	*z*	−40	160
23/50	3.0	*z*	−50	100
60 °C water	8.2	*z*	−50	100

**Table 3:** DSC experiments: overview.

Conditioning	$M_{\mathrm{eq}}$ (%)	$T_{\min}$ (°C)	$T_{\max}$ (°C)
Dry	0.0	20	100
23/50	3.0	−10	80
80 °C water	8.1	−50	30

**Table 4:** DSC: glass transition temperature (10 K min^−1^).

Conditioning	$T_{g}$ (°C)
Dry	58
23/50	24
80 °C water	−24

**Table 5:** YOUNG’s moduli and POISSON’s ratios for different conditioning states tested at 23 °C.

Conditioning	$M_{\mathrm{eq}}$ (%)	Young’s Modulus (GPa)	Poisson’s Ratio
Dry	0.0	2.885	0.391
23/50	3.0	1.235	0.465
60 °C water	8.2	0.598	0.478

**Table 6:** CME for 2 mm plate-shaped specimens.

Measurement	CME β (kg^−1^ m^3^)
*x*	1.814 × ^−4^
*y*	1.823 × ^−4^
*z*	3.681 × ^−4^
Iso. from x,y,z	2.436 × ^−4^
Iso. from buoyancy	2.434 × ^−4^

**Table 7:** CTE for dry (4×4×4)$m^{3}m$ cube.

Temperature *T* (°C)	Secant CTE α (K^−1^)
−20	71.18 × ^−4^
30	77.57 × ^−6^
60	86.73 × ^−6^
100	107.9 × ^−6^
130	121.5 × ^−6^

## Data Availability

The data presented in this study are available on request from the corresponding author.
